# Total hip arthroplasty for destructive septic arthritis of the hip using a two-stage protocol without spacer placement

**DOI:** 10.1007/s00402-021-03981-2

**Published:** 2021-06-07

**Authors:** Christian Hipfl, Daniel Karczewski, Jakub Oronowicz, Matthias Pumberger, Carsten Perka, Sebastian Hardt

**Affiliations:** Department of Orthopaedic Surgery, Center for Musculoskeletal Surgery, Charité-Universitätsmedizin Berlin, Charitéplatz 1, 10117 Berlin, Germany

**Keywords:** Septic arthritis, Hip, Resection arthroplasty, Two-stage, Spacer, Total hip arthroplasty, Periprosthetic infection

## Abstract

**Introduction:**

The optimal treatment of patients with a degenerative joint disease secondary to an active or chronic septic arthritis of the hip is unclear. The aim of the present study was to report on our experience with two-stage total hip arthroplasty (THA) using a contemporary treatment protocol without spacer insertion.

**Materials and methods:**

Our prospective institutional database was used to identify all patients with degenerative septic arthritis treated with a non-spacer two-stage protocol between 2011 and 2017. Clinical outcomes included interim revision, periprosthetic infection (PJI) and aseptic revision rates. Restoration of leg-length and offset were assessed radiographically. Modified Harris hip score (mHHS) were obtained. Treatment success was defined using the modified Delphi consensus criteria. Mean follow-up was 62 months (13–110).

**Results:**

A total of 33 patients with a mean age of 60 years (13–85) were included. 55% of the cohort was male and average Charlson Comorbidity Index (CCI) was 3.7 (0–12). 21 patients (64%) had an active/acute infection and 12 patients (36%) were treated for chronic/quiescent septic arthritis. Overall, 11 patients (33%) had treatment failure, including 5 patients who failed to undergo THA, 2 interim re-debridement for persistent infection, and 4 patients who developed PJI after an average of 7 months (0.3–13) following THA. The most common identified pathogen was *Staphylococcus aureus* (42.4%). No aseptic revision was recorded following THA. Leg-length and offset were successfully restored. Mean mHHS improved from 35.2 points to 73.4 points.

**Conclusion:**

Two-stage THA without spacer placement is a viable treatment option for destructive septic arthritis of the hip, demonstrating comparable rates of infection control and functional outcome. However, definitive resection arthroplasty is not uncommon in these often critically ill patients.

## Introduction

Septic arthritis of the native hip is a rare, but potentially devastating disease which can lead to irreversible damage of the affected joint [1, 2]. Its heterogenous etiology and clinical presentation pose a challenge for both diagnosis and treatment. Risk factors for the emergence of a bacterial arthritis include diabetes, rheumatoid arthritis, intravenous drug abuse, chronic liver disease, cancer and any cause of immunodeficiency [3–6]. Septic arthritis can manifest within many different contexts: patients with recalcitrant infection in otherwise healthy joints, patients with pre-existing degenerative joints who develop septic arthritis, and patients who develop osteoarthritis after septic arthritis. Due to this multifactorial pattern of presentation, there are still no guidelines or consensus on the treatment algorithm for these patients [7].

While arthroscopic or open debridement with intravenous antibiotics for acute septic arthritis of the hip with its retained anatomic structures is an established, initial management [3, 8], the optimal therapy for recalcitrant and destructive septic hip arthritis remains controversial. A primary total hip arthroplasty (THA) is a risky choice in the face of a lingering suspicion of infection, because persistent infection is likely to result in periprosthetic infection (PJI) [9–11]. In the past years, some reports have been published about the concept of using a two-stage procedure for the treatment of these patients [11–17]. Two-stage exchange arthroplasty with antibiotic spacer placement has become a common treatment for PJI [18] and has also been described for the treatment of septic arthritis [11, 13–17]. However, the local antimicrobial effects of the spacer seem to play a negligible role [19] and in the last years high rates of spacer-related complications have been reported, including spacer dislocation, spacer migration, spacer breakage and femur fracture [20–23]. Moreover, in the setting of a native joint, infection only involves articular cartilage, subchondral bone and intracapsular soft-tissue, and spacer insertion might lead to a spread into an otherwise intact, femoral medullary cavity. Finally, patients with reluctant bacterial hip arthritis are often multimorbid patients unfit or unwilling to undergo second-stage surgery and resection arthroplasty may represent the definitive treatment [11, 15]. These considerations led us to largely dispense with hip spacers in this challenging patient population. However, a major concern of a non-spacer treatment is that muscle contractures hamper reimplantation and hence leading to leg-length discrepancy and worse functional outcomes [24]. However, only few data exist and some literature dates back in the 20th century [12, 25].

Therefore, the aim of the present study was to evaluate the results of a contemporary two-stage protocol without spacer placement in the treatment of destructive septic arthritis of the hip.

## Patients and methods

### Study design and patient selection

Following institutional review board approval, we retrospectively reviewed our prospectively maintained institutional database for the period October 2011 to November 2017 to identify all consecutive patients who underwent a two-stage THA for an acute or chronic native hip joint infection. Inclusion criteria were patients who underwent the resection arthroplasty at our institution and had not undergone a spacer implantation. Exclusion criteria were any violation of the standardized treatment protocol.

Medical records were reviewed for all details on demographics, comorbidities, age-adjusted Charlson Comorbidity Index (CCI) [26], mode of presentation, laboratory data including C-reactive protein (CRP), intraoperative findings and postoperative follow-up.

### Diagnosis of infection

All patients presented with an acute or chronic painful hip and clinical suspicion of infection. A standardized preoperative diagnostic work-up included physical examination, plain radiographs and laboratory tests, including serum C-reactive protein (CRP). 11 of the 33 patients (33%) had a prior surgical intervention for infection either at our institution or at the referring institution. Percutaneous synovial fluid aspiration guided by fluoroscopy was able to detect a microorganism in 8 of the 33 patients (23%) prior to resection arthroplasty.

**Fig. 1:**
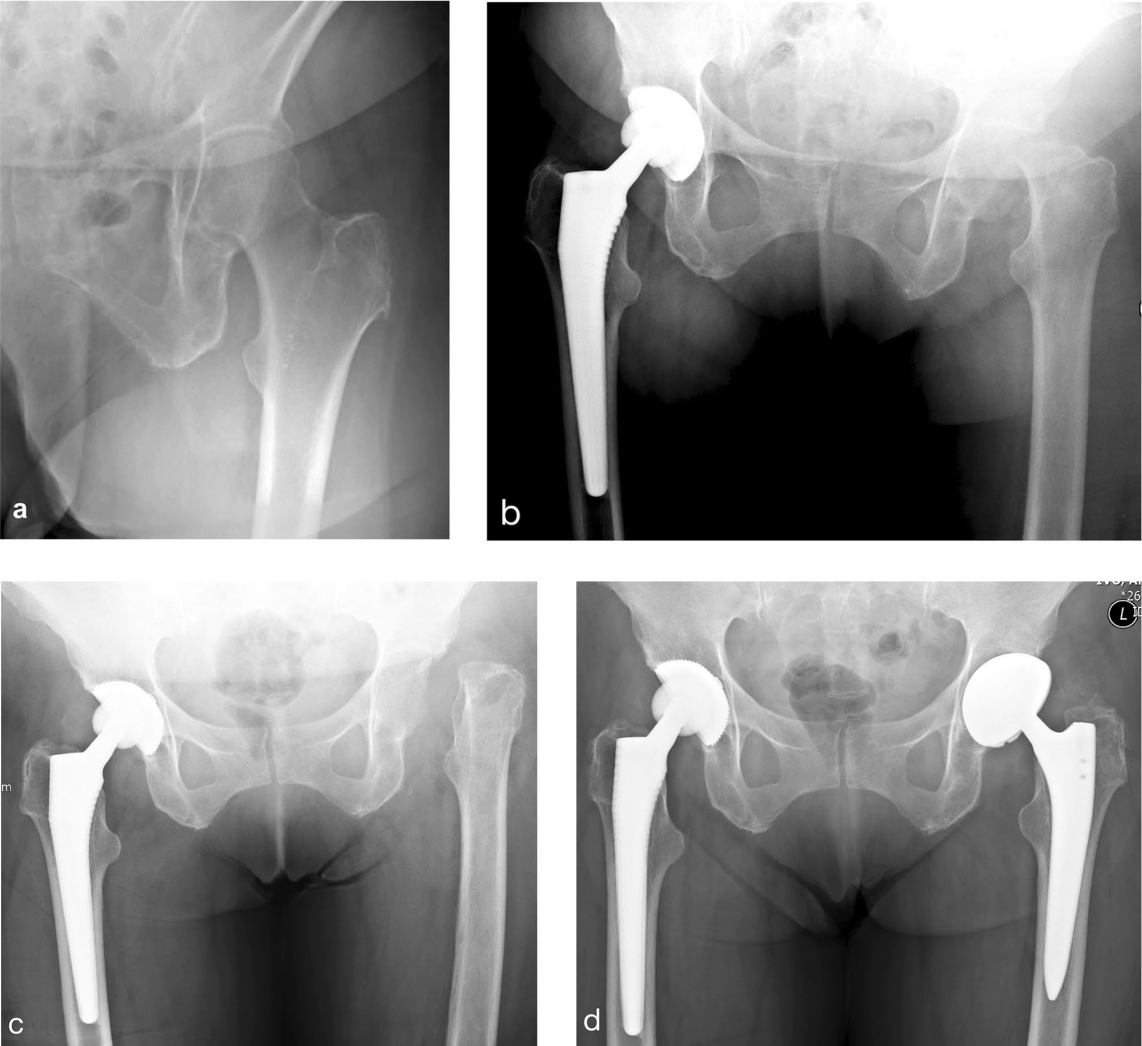
Radiographs of a 79-old female patient with destructive septic arthritis of the left hip. **a** Anteroposterior view 5 months before clinical presentation. **b** Preoperative radiograph prior to two-stage total hip arthroplasty (THA). **c** Postoperative radiograph following resection arthroplasty without spacer insertion. **d** Postoperative radiograph showing the definitive THA after an interim period of 4 weeks using a highly porous acetabular shell and an extensively porous-coated femoral stem. Leg-length was successfully restored. At latest follow-up, the patient showed no signs of PJI and had a modified Harris hip score of 63 points

Septic arthritis of the hip was diagnosed when clinical suspicion (painful, red, hot, swollen and/or restricted joint) plus at least one of the following criteria was present: confirmatory microbial growth (in synovial fluid, tissue and/or blood culture), increased leukocyte count in synovial fluid (> 50.000/μl or > 90% granulocytes) or positive histopathology [6, 27]. All patients in the present study had a degenerative joint disease or osseous destruction secondary to a previously treated acute septic arthritis and presented with either active infection or chronic changes secondary to a previous infection (Fig. 1a, b). An acute/active infection was defined as cases with new onset symptoms and/or who had been previously treated with antibiotics and/or irrigation and debridement, within 3 weeks before first-stage surgery. A chronic/quiescent infection was defined as cases with chronic symptoms and/or who had previous septic arthritis treatment or non-arthroplasty treatment with history of infection, greater than 3 weeks before the resection arthroplasty.

### Surgical treatment

During the first-stage procedure, all foreign material was removed and all infected tissue was debrided, while ensuring preservation of the abductors. The femoral neck was osteotomized in routine fashion as during a primary THA. Inflamed synovial lining around the acetabulum was resected and the acetabulum was sequentially reamed to remove the remaining cartilage. Explanted internal fixation devices were sent for sonication (if present), synovial fluid was aspirated (if present) and a minimum of five periprosthetic tissue samples were collected for microbiological analysis. This was followed by a thorough irrigation and debridement (I&D) of bone and soft-tissue using a polyhexanide-containing solution. The femoral canal was kept closed and no cement spacer was implanted (Fig. 1c). The wound was thoroughly irrigated and closed routinely in layers over passive drains. The mean operative time of first-stage surgery was 90 min (35–180).

Drains were removed after 48 h and patients were mobilized with the help of a walker or crutches under toe-touch weight bearing. The individual prosthesis-free interval was determined by the causing pathogen, the clinical course and patient’s general health condition. THA reimplantation was performed only when the local status was satisfactory (surgical wound healed, no drainage, redness or increased swelling), laboratory signs of infection control (continuously decreasing C-reactive protein) were present, and the general status of the patient was suitable. Any evidence of persistent infection led to interim re-debridement.

The second-stage surgery with definitive THA was predominantly performed using cementless components through an anterolateral approach (Fig. 1d). In 2 of 28 hips (7.1%) acetabular and femoral components were cemented. Tapered rectangular femoral stems were used in all uncemented THA. Modular porous coated shells were used in 15 hips and highly porous, Trabecular Metal (Zimmer Biomet) shells were utilized in 11 hips. Standard highly cross-linked polyethylene acetabular liners were used in all hips. Before insertion of the THA, microbiological samples were obtained and thorough I&D was performed in the same fashion as during the first-stage surgery. The mean operative time of the second-stage surgery was 120 min (57–184).

### Antimicrobial treatment

The antimicrobial therapy was selected by infectious disease specialists according to a previously established treatment protocol, which is in line with our protocol for PJI [28, 29] and have been used in several clinical outcome studies [30–32]. After the first-stage surgery, broad-spectrum, intravenous antibiotics were initiated postoperatively and administered for 2 weeks followed by oral antibiotics until definitive THA. Oral antibiotics were selected according to high oral bioavailability, osseous penetration and susceptibility testing of the causative organism. Between the first-stage resection arthroplasty and definitive THA all patients received a continuous systemic antimicrobial treatment. No antibiotic holiday or diagnostic hip aspiration were performed prior to THA insertion [33]. The minimum duration of antimicrobial therapy between the first and second stage surgery was 2 weeks with a mean duration of 9.7 weeks (range 2–32 weeks).

After second-stage THA, intravenous antibiotics were administered for two weeks postoperatively followed by oral antibiotics for a minimum of four weeks. In cases of confirmatory microbiological results at second-stage THA (≥ 2 positive specimens, polymicrobial growth, or ≥ 1 positive specimen, if the isolated microorganism was the same as the initial infecting pathogen or a new highly virulent organism), antimicrobial treatment was prolonged from 6 to 12 weeks after THA. Treatment with biofilm-active antibiotics (e.g. rifampicin or quinolones) was started after THA reimplantation when the wound was dry to avoid the emergence of resistant strains [34]. The total duration of antimicrobial treatment, from resection arthroplasty until the end of antimicrobial therapy after definitive THA, was 16.3 weeks on average (range 8–38 weeks).

### Treatment outcome

Patients were evaluated during follow-up examinations in our outpatient clinic at 6-week, 3-month and 1-year intervals. Medical records were reviewed to identify revisions performed for the occurrence of PJI and other complications. Infectious diseases physicians were consulted to help identify reinfections. Treatment outcome was judged according to the modified Delphi international multidisciplinary consensus criteria of treatment for PJI [35] as follows: (1) infection eradication, characterized by a healed wound without sinus tract, drainage or significant pain, and no infection recurrence caused by any organism strain; (2) no subsequent surgical intervention for infection after resection arthroplasty; (3) no infection-related mortality; (4) no long-term antimicrobial suppression therapy (duration > 6 months); and (5) no failure to THA.

### Leg-length restoration and functional outcome

Preoperative and postoperative radiographs were analyzed to assess the leg-length and offset. Leg-length discrepancy was measured via vertical height difference between the interteardrop/interpubic tubercle lines and the lesser tubercle line; offset discrepancy was calculated as the difference between the THA offset and contralateral offset.

Functional outcome was assessed for all surviving patients, which did not require revision surgery following second-stage THA, calculating the modified Harris Hip Score (mHHS) [36, 37] preoperatively and at the latest follow-up.

### Statistical analysis

Descriptive statistics are reported as number (percentage) or mean (range, standard deviation), as appropriate. Continuous variables were compared using the Mann–Whitney *U* and *t* test, categorical variables using the Chi-square test. A *p* value < 0.05 was considered statistically significant. Calculations were performed using SPSS version 25 software (SPSS Inc., Chicago, IL, USA).

## Results

### Patient demographics

A total of 33 patients (33 hips) treated for acute/active infection (*n* = 21, 64%) or chronic/quiescent infection (*n* = 12, 36%) were included. The mean age at the time of resection arthroplasty was 60.4 years (13–85) and mean body mass index (BMI) was 27 kg/m^2^ (19–33). The majority of patients (*n* = 18, 55%) were male and the mean age-adjusted Charlson Comorbidity Index (CCI) was 3.7 (0–12). 17 out of 33 patients (52%) underwent a prior surgery of any kind with an average of 2.4 prior surgeries per case (1–5). 10 out of 33 patients (30%) had previous internal fixation of a femoral neck (*n* = 4) or acetabular fracture (*n* = 6). Six patients (18%) had systemic sepsis at the time of presentation. Concomitant spondylodiscitis and infection of another joint at the same time was present in 5 patients (15%) and 2 patients (6%), respectively. An abscess of the psoas muscle was present in 10 cases (30%). The mean interim period from resection arthroplasty to definitive THA was 9.7 weeks (2–32). All demographic data and operative characteristics stratified by whether patients presented with an active or chronic/quiescent hip joint infection are summarized in Tables 1 and 2, respectively.

**Table 1:** Demographics

Variable	Acute/active infection (n = 21)	Chronic/quiescent infection (*n* = 12)	All hips (*n* = 33)	*p* value
Age at first-stage (years)	65.3 ± 15.4	51.7 ± 21.4	60.4 ± 18.7	**0.043**
Sex (M:F) (*n*)	10/11	8/4	18/15	0.290
BMI (kg/m^2^)	26.6 ± 3.8	26.9 ± 4.4	26.7 ± 4.5	0.808
ASA score	2.5 ± 0.6	2.2 ± 0.6	2.4 ± 0.6	0.316
CCI	4.5 ± 2.9	2.2 ± 1.8	3.7 ± 2.7	**0.018**
Diabetes mellitus (*n*)	5 (24%)	2 (17%)	7 (21%)	0.692
Rheumatoid arthritis (*n*)	1 (5%)	1 (8%)	2 (6%)	0.679
Cancer (including past cases) (*n*)	8 (38%)	2 (17%)	10 (30%)	0.198
Other immunodeficiency (*n*)	0 (0%)	1 (8%)	1 (3%)	0.179

Bold—the significance level was *p* < 0.05

*BMI* body mass index, *ASA* American Society of Anaethesiologists, *CCI* Charlson Comorbidity Index

**Table 2:** Operative characteristics

Variable	Acute/active infection (*n* = 21)	Chronic/quiescent infection (*n* = 12)	All hips (*n* = 33)	*p* value
Posttraumatic cases (*n*)	3 (14%)	7 (58%)	10 (30%)	**0.008**
Prior open surgical procedures (*n*)	8 (38%)	9 (75%)	17 (52%)	**0.041**
Revision for infection (*n*)	5 (24%)	6 (50%)	11 (33%)	0.125
Psoas abscess present (*n*)	10 (48%)	0 (0%)	10 (30%)	**0.004**
CRP before first-stage	127.9 ± 90.4	18.3 ± 34	88 ± 91.5	**0.001**
Duration of first-stage surgery (min)	88.7 ± 36.5	93.5 ± 38.9	90 ± 36.9	0.775
Weeks between stages	11 ± 7.0	8.1 ± 5.9	9.7 ± 6.6	0.163
Interim re-debridement for infection (*n*)	1 (5%)	1 (8%)	2 (6%)	0.679
Interim re-debridement for hematoma (*n*)	2 (10%)	0 (0%)	2 (6%)	0.270
No definitive THA	5 (24%)	0 (0%)	5 (15%)	0.133
CRP before second-stage	16.9 ± 15.8	5.5 ± 4.2	12.09 ± 13.4	0.133
Cemented components at second-stage				
Acetabular cemented (*n*)	2 (10%)	0 (0%)	2 (16%)	0.270
Femoral cemented (*n*)	2 (10%)	0 (0%)	2 (16%)	0.270
Duration of second-stage (min)	150.5 ± 77.2	113.0 ± 23.0	134.5 ± 62.3	0.163

Bold—the significance level was *p* < 0.05

*CRP* C-reactive protein, *THA* total hip arthroplasty

### Treatment outcome

**Fig. 2:**
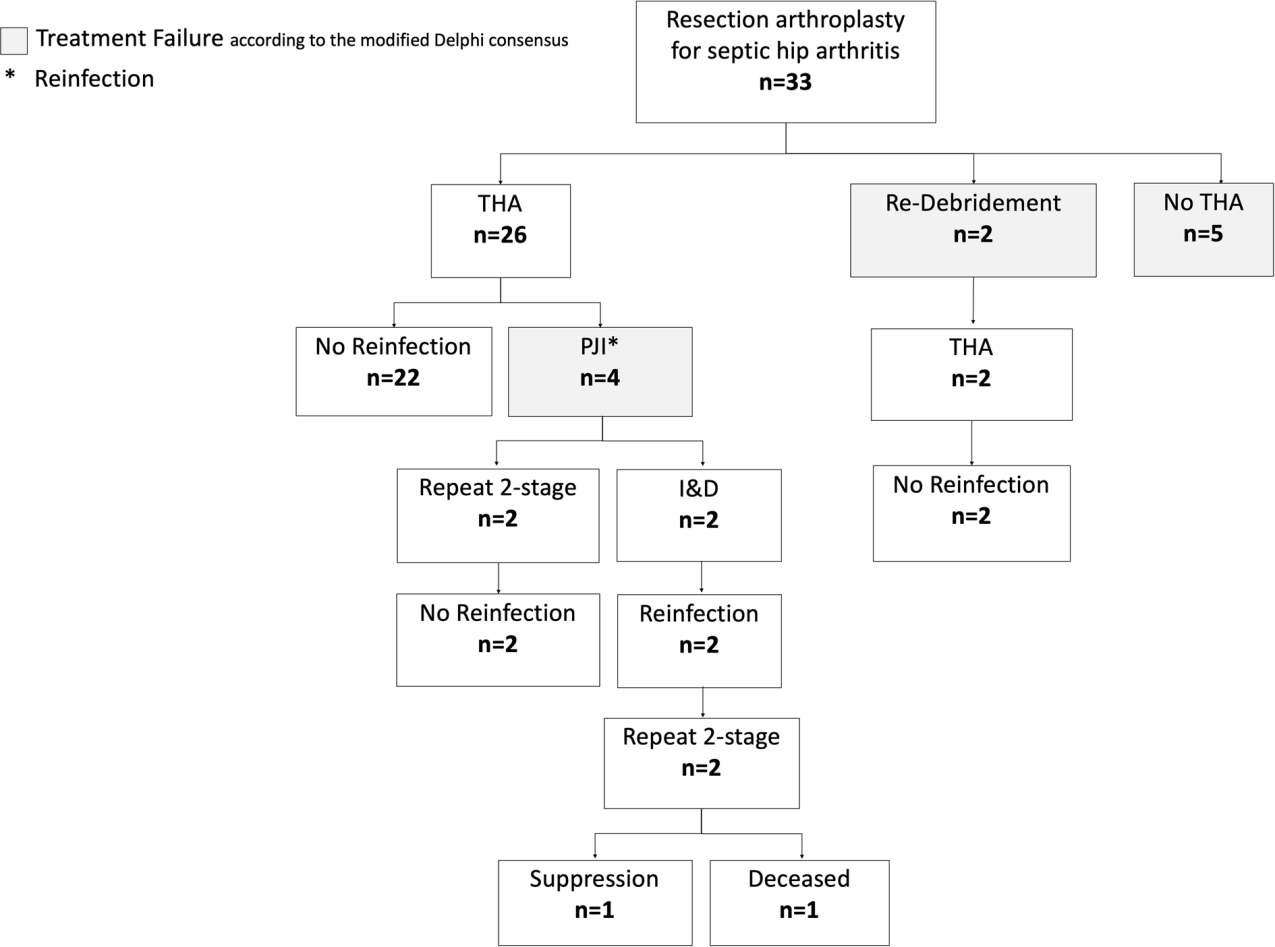
Flowchart outlining the treatment outcome of all patients included in the study

In our cohort of 33 patients undergoing non-spacer two-stage THA for septic hip arthritis with a mean follow-up of 62 months (13–110), 11 patients (33%) had treatment failure according to the modified Delphi international multidisciplinary consensus (Fig. 2). 4 patients (12%) developed PJI after second-stage THA, with 1 having growth of the same organism and 3 having growth of a newly detected (*n* = 2) or different (*n* = 1) organism at the subsequent surgical intervention. 2 patients (6%) had persistent infection in the interim period and underwent repeat I&D after 13 and 14 days, respectively. 5 patients (15%) failed to undergo definitive THA.

Overall, 4 of the 28 cases (14.2%) undergoing THA reimplantation had experienced PJI after a mean of 7.1 months (0–13). All four patients with PJI underwent surgical intervention; with two undergoing a two-stage exchange arthroplasty without relapse and two undergoing I&D with head-and-liner exchange. The latter two failed and were subsequently treated with a two-stage exchange THA, whereas one patient with a severe immunological defect (hypogammaglobinemia) required long-term antibiotic suppressive therapy. The other patient had a history of pelvic osteomyelitis secondary to infective endocarditis and suffered from polymicrobial PJI including Candida species. This patient died in the early postoperative course following the two-stage exchange arthroplasty. We found no significant associations between demographic variables or operative characteristics and the occurrence of PJI. The characteristics of all patients who developed PJI are shown in Table 3.

**Table 3:** Information summary of all patients who developed PJI

Case	Type of infection	Age (years)	Sex	CCI	Isolate at first-stage	Interval (weeks)	Isolate at second-stage	Time to PJI (months)	Isolate at revision for reinfection	Follow-up (months)	Final outcome
1	Chronic/quiescent infection	54	Male	3	Negative	2.9	Negative	4.9	*S. dysgalactiae, S. hominis*	46.3	No reinfection after subsequent two-stage
2	Chronic/quiescent infection	29	Male	4	*C. difficile*	5.0	*C. difficile*	12,6	*C. difficile, S. epidermidis*	45.0	Antibiotic suppression after subsequent two-stage
3	Acute/active infection	67	Female	5	*S. lugdunensis, M. luteus*	12.6	Negative	0.3	*C. albicans, S. epidermidis, E. coli, E. faecalis, E. cloacae*	17.9	Deceased
4	Acute/active infection	64	Female	4	Negative	6.1	Negative	10.7	*S. lugdunensis, S. epidermidis, F. magna*	21.7	No reinfection after subsequent two-stage

*CCI* Charlston Comorbidity Index, *PJI* periprosthetic joint infection

### Microbiological findings

The microorganism leading to septic arthritis were identified in 23 cases (69%). Pathogens were detected by preoperative joint aspiration, intraoperative samples or by referencing past charts from patients who presented with chronic/quiescent infections. The most commonly detected microorganism was *Staphylococcus aureus* (42.4%) followed by coagulase-negative *Staphylococcus* (12.1%) and *Cutibacterium acnes* (12.1%). Details of all microorganisms isolated preoperatively and at the first-stage surgery are shown in Table 4.

**Table 4:** Microorganism frequency (includes preoperative and intraoperative cultures)

Isolated microorganism (*n*)	Acute/active infection (*n* = 21)	Chronic/quiescent infection (*n* = 12)	All hips (*n* = 33)	*p* value
Gram-positive bacteria	14	8	22	0.999
*Staphylococcus aureus*	11	2	13	**0.043**
Coagulase-negative *Staphylococcus*	3	3	6	0.443
*Cutibacterium *spp.	0	3	3	**0.016**
Gram-negative bacteria	0	0	0	–
Negative culture	7	4	11	0.999
Polymicrobial	3	2	5	0.854

Bold—the significance level was *p* < 0.05

At second-stage THA, 3 of 28 hips (11%) presented with confirmatory microbiological cultures and among those, one patient suffered from PJI caused by the same pathogen. Both patients who required an interim re-debridement for infection persistence showed growth of the initial infecting pathogen (*Staphylococcus aureus*). Among the latter, no PJI occurred after THA insertion. Overall, 3 of the 4 hips (75%) with infection recurrence (i.e. PJI) showed growth of newly detected or different organisms than in the previous cultures.

### Leg-length restoration and functional outcome

Leg-length and offset was restored with a mean postoperative leg-length discrepancy of − 1.0 mm and offset discrepancy of − 1.3 mm compared to preoperatively − 13.9 mm and − 3.2 mm, respectively (*p* < 0.01 and *p* = 0.061). mHHS were available for 19 (79%) of 24 patients who were alive and free of revision at the latest follow-up. The mean mHHS score improved from 35 points preceding first-stage surgery to 72 points at final follow-up (*p* < 0.01). 10 patients walked without support, three patients used a cane for long walks, five patients used a cane most of the time and one patient with a BMI of 33 kg/m^2^ was only able to do short distances using two crutches.

### Complications

None of the patients have experienced a mechanical complication in the interim period. Two patients required reoperation for postoperative hematoma following first-stage surgery. No aseptic revision was recorded following second-stage THA. Overall, 20 of the 28 patients (71%) undergoing the entire two-stage protocol did not have any complication, reoperation, or postoperative sequela.

## Discussion

Septic arthritis of the hip remains a challenging diagnosis. Acute infections of the undamaged joint are conventionally managed with debridement and antibiotics. However, patients frequently present with recalcitrant infection or secondary degenerative joint disease related to an acute or chronic septic arthritis. Most authors have advocated a two-stage exchange total hip arthroplasty with the use of antibiotic-loaded spacers in the interim period to treat such cases [11–17]. However, spacer utilization bears the risk of mechanical failure during the interim period and patients are often medically unfit to undergo second-stage surgery. To the best of our knowledge, the underlying study reports the largest cohort of patients with destructive septic arthritis of the hip treated with a contemporary two-stage protocol without spacer placement. We found comparable rates of infection control and functional outcome. A considerable number of patients, however, failed to undergo definitive THA.

Prior studies reporting on two-stage THA for the treatment of native septic hips offer a broad range of diagnostic criteria and outcome measures regarding successful infection control [11–17]. Due to the heterogenous baseline cohorts and varying treatment protocols, it is difficult to compare the results. The definition of infection varies between the studies, as consensus on diagnostic criteria and treatment algorithms are still lacking for native hip joint infections, with the exception of the “Kocher criteria” for pediatric septic hip arthritis [38]. To date, there are no guidelines when to perform a resection arthroplasty. However, a delayed presentation beyond three weeks seems to be a strong predictor of the need to sacrifice the joint [1] while in the coexisting degenerative joint the need for joint replacement is evident. In the underlying study, we used the same rationale as for chronic PJI and performed a two-stage exchange arthroplasty utilizing an equivalent treatment protocol [28, 29] Moreover, a strict definition of treatment failure was applied using the modified Delphi international multidisciplinary consensus criteria, which has become the contemporary outcome measure in PJI studies [35].

In our cohort, more than 60% of the patients had an acute/active infection, of which almost the half had a concomitant psoas abscess and more than a quarter had systemic sepsis at time of presentation. Patients with an acute infection were significantly older and had a significantly higher CCI compared to patients that presented with chronic infections. While in terms of interim revision and PJI rates, we found no difference between acute and chronic infections, all patients who failed to undergo second-stage THA presented with an acute infection.

Our study demonstrated an overall treatment failure of 33% according to the modified Delphi international multidisciplinary consensus criteria. The overall rate of recurrence of infection after THA was 14% at a mean follow-up of 62 months. This figure is toward the upper end of PJI rates previously reported for two-stage THA, ranging between 0 and 15% [11–17]. Among the infection recurrences in our cohort, three-fourth were related to pathogens that were newly detected or differed from the original infection. There are several possible explanations for this. First, the pathogen was missed during the two-stage THA. One-third of our cohort was culture-negative, which is consistent with the literature [2, 17]. Particularly, when searching for less-virulent or atypical organisms and in patients with preceding antimicrobial treatment, standard cultures are frequently false negative [6, 39]. This emphasizes the need for more sensitive diagnostic methods, such as PCR [40]. Second, it is possible that the patient had polymicrobial septic arthritis and the additional species were not detected during two-stage THA [41]. Finally, the newly detected bacterial species can represent a new infection, introduced during the second-stage reimplantation surgery or due to hematogenous seeding (e.g. *Streptococcus dysgalactiae*) [42, 43]. The only PJI caused by the previously isolated pathogen (*Clostridium difficile)* can be attributed to a severe immune deficiency and infection was ultimately controlled by antibiotic suppression therapy.

In the underlying study, 15% did not undergo second-stage surgery due to a general poor health status. While patients who fail to undergo second-stage THA were largely overlooked in previous studies [12, 13, 16, 17], the attrition rates reported by other authors ranged between 0 and 29% [11, 14, 15]. These relatively high rates of non-completed second-stage cases may have resulted in an overestimation of some previously reported success rates.

The present study found that 2 of 33 patients (6%) required re-debridement for persistent infection before definitive THA. These figures are comparable to the literature which shows spacer exchange rates ranging between 0 and 23% [11–17]. The main rationale behind our non-spacer two-stage exchange protocol was the high risk of spacer-related mechanical complications reported in PJI cohorts [11–17]. In cohorts treated for septic native hips, spacer-related issues, such as spacer dislocation or fracture, were reported in up to 23% [14, 15]. In the underlying study, no mechanical complications were observed during the interim period.

Cementless THA was performed in over 90% of the cases and no aseptic revision was required after a mean follow-up of 62 months. This is at the lower end of aseptic revision rates reported in the literature, ranging between 0 and 20% [11–17]. We believe that cementless fixation of THA is practicable in the majority of cases, especially when the femoral medullary cavity is kept intact during the resection arthroplasty.

Finally, a concern of utilizing temporary resection arthroplasty as an interim strategy is limp shortening and muscle contracture making reimplantation technically more challenging, especially leg-length restoration. In the underlying series; however, leg-length and offset were successfully restored and final clinical outcome scores improved significantly to values that are comparable to previous studies reporting on two-stage THA with spacer placement [11, 13–17].

The authors acknowledge the limitations of this study. First, its retrospective study design and small patient population are subject to associated biases common to these studies. Second, although we have used a standardized two-stage protocol, several variables, including degree of debridement, length of interval, implant selection for reimplantation had minor variations according to surgeon and infectious disease specialist preference. Third, we did not evaluate the perioperative morbidity and patient function or satisfaction during the interim period. Finally, a prospective study comparing two-stage THA with and without the use of hip spacers including the effect on the perioperative morbidity and patient satisfaction would provide additional valuable information.

In conclusion, this study shows that two-stage THA without spacer placement is a viable treatment option to manage destructive septic arthritis of the hip. Patients treated for septic arthritis of the hip were often medically unfit and resection arthroplasty not seldomly represented the definitive treatment. Most reinfections following THA were related to newly detected or different pathogens. Cementless THA showed good survivorship at the mid-term, with comparable functional outcomes and leg-length restoration. Future studies should investigate the optimal interim length and whether in more remote/quiescent infections a direct THA would be sufficient.
